# Multi-Detection of Veterinary Medicines in Animal Feed for Production: A Review

**DOI:** 10.3390/antibiotics14121233

**Published:** 2025-12-07

**Authors:** Ana Lúcia Lopes, Marta Leite, Maria Beatriz P. P. Oliveira, Andreia Freitas

**Affiliations:** 1 Faculty of Pharmacy, University of Porto, Rua de Jorge Viterbo Ferreira 228, 4050-313 Porto, Portugal; up202408523@up.pt (A.L.L.); beatoliv@ff.up.pt (M.B.P.P.O.); 2 National Institute for Agrarian and Veterinary Research (INIAV), Rua dos Lagidos, Lugar da Madalena, 4485-655 Vila do Conde, Portugal; marta.leite@iniav.pt; 3 Associated Laboratory for Green Chemistry (LAQV) of the Network of Chemistry and Technology (REQUIMTE), Praça Coronel Pacheco n°15-6°, 4050-453 Porto, Portugal

**Keywords:** antibiotics, animal feed, veterinary residues, liquid chromatography, mass spectrometry

## Abstract

**Background/Objectives**: The inappropriate use of veterinary medicines in feed for food-producing animals can compromise food safety. Intensive animal production is associated with the inappropriate use of antibiotics in feed, at subtherapeutic concentrations, to promote animal growth. It is therefore crucial to develop an effective multi-detection method to ensure that this feed complies with the requirements of European Commission Regulations. This control is essential to ensure consumer protection, as adequate supervision contributes to reducing antimicrobial resistance, a growing concern worldwide. **Methods**: A literature search was conducted using scientific databases, namely PubMed, ScienceDirect, Scopus and Google Scholar, as well as European Union Regulations. **Results**: It was observed that the most used standard solution solvents are methanol, acetonitrile, ultrapure water, or mixtures of these solvents. For extraction, the most frequently used solvents include trichloroacetic acid combined with McIlvaine buffer or with acetonitrile, and acetonitrile or methanol combined with formic acid or with ethylenediaminetetraacetic acid disodium (Na_2_EDTA). For extraction and purification of the analyte, several steps were verified, such as solid-phase extraction (SPE), dispersive solid-phase extraction (d-SPE), liquid–liquid extraction (LLE), Quick, Easy, Cheap, Effective, Rugged, and Safe (QuEChERS), protein precipitation through freezing and dilution prior to analysis. Liquid chromatography coupled with mass spectrometry is the preferred choice, especially for multiple detection methods. **Conclusions**: Based on this data, the foundation is established for the development of an appropriate method for the simultaneous extraction of multiple classes of antibiotics, which is applicable to feed different food-production animals.

## 1. Introduction

In recent years, developing countries have seen an increase in their economic power. Consequently, the global demand for food of animal origin has grown, and, as the income of the population in these countries increases, there is greater consumption of animal products, which requires an increased use of agricultural resources [1,2]. A diet based on food of animal origin requires substantially more agricultural production than plant-based diets. It is calculated, for example, that one kilogram of beef per year requires approximately 20.9 square meters of land, a figure around 15 times greater than the area of land used to plant cereals in the same proportion and time [3]. The Livestock sector contributes about 14% of global greenhouse gas emissions [4].

The animal sector occupies approximately 40% of agricultural land globally. This intensifies the competition for land use between crop production for human consumption and animal feed production. Various strategies have been implemented to minimise this competition and reduce environmental impact. These include reducing the consumption of food of animal origin, especially in high-income countries, since they have greater access to nutritional education and new sources of protein, such as insects and algae [5]. In addition, the recovery of by-products from human food for animal feed, as well as the reuse of inedible by-products for the production of fertilisers, are sustainable solutions. Biological treatment of by-products, such as straw, through the action of fungi, can improve the nutritional value of animal feed, optimising the use of resources and contributing to the circular economy [2,6,7]. These approaches contribute to reducing land conflicts and cooperate towards an effective circular food system, promoting the reduction in environmental impact and, consequently, the improvement of human health [5].

Despite the strategies for more sustainable agriculture practices, intensive animal production continues to increase, as it is the fastest way to meet the nutritional needs of the world’s population in terms of protein and minerals [6,8]. The growing demand for animal products has reinforced the prevalence of intensive production over extensive production. One of the main factors explaining this competitive advantage is the high productivity of intensive industries, which they combine with the ability to establish long-term sales contracts at fixed prices with processing companies. This commercial stability gives intensive companies significant advantages over farming systems that sell less, such as extensive production [9].

The intensive animal production system is characterised by the high density of animals in a restricted space, in which the animals are kept in confinement, and is associated with the use of growth promoters. This system uses fast-growing breeds and automated operations [10]. Advanced technologies such as embryo transfer and artificial insemination are used to increase productivity [6]. On the other hand, extensive production promotes the welfare and quality of animal products. Within this system, there are various types, including antimicrobial-free farming and organic specification, in which production is restricted to the use of antibiotics and 100% organic feed [10].

For the purpose of treating diseases or prevention infection spread, antibiotics can be administered to animals orally, parenterally or topically. When administered by injection, these drugs tend to accumulate in fatty tissue, even after the animal has been slaughtered [11,12]. The most common form of oral administration of veterinary medicines is through medicated feed, which requires a homogeneous mixture between the feed and the medicines [13]. The presence of these residues is more widely used than liquid feed, as it has a greater capacity for conservation over time, as well as being easier to transport and distribute [14].

In the case of fish, administration can be oral, whereby antibiotics are added to the water or mixed into the feed, by injection, which is a difficult treatment, or by bathing. Both oral and bathing methods result in antibiotic residues contaminating the aquatic environment, which leads to the transfer of bacterial resistance genes in these ecosystems. The situation is exacerbated when resistance genes are exchanged between aquatic and terrestrial ecosystems, which can harm human and animal health [15].

Prophylaxis is a preventative strategy that involves administering low doses of antibiotics through animal feed over a prolonged period. Metaphylaxis, on the other hand, is characterised by the administration of high doses of antibiotics over a short period, aimed at preventing the transmission of infectious diseases among healthy animals that come into contact with infected animals. Both approaches aim to prevent infectious diseases, but exposing a group of animals to a common concentration of antibiotics favours the development of antimicrobial resistance [16,17].

Antimicrobial resistance (AMR) is generally more prevalent in intensive production than in extensive production. However, animals in extensive production are not exempt from this problem, since access to the external environment exposes animals to potential dangers of microbiological contamination, which increases the risk of disease and, consequently, the need for antimicrobial treatments, favouring the emergence and spread of resistant microorganisms [10]. On the other hand, intensive production has higher direct or indirect contact with animals or consumption of food, through measures such as environmental and sanitary control, which contribute to disease prevention and can minimise the need to use antibiotics, which reduces the transmission of AMR [8,10].

Thus, antibiotic resistance is a global public health concern in both production systems. However, in intensive production, this problem is more pronounced, since the high population density and confinement of animals are favourable to the transmission of resistant pathogens between animals and humans [18]. Direct or indirect contact with animals or consumption of food of animal origin can transmit microorganisms resistant to certain antibiotics [16,19]. Although antibiotics can be administered in livestock farming to treat diagnosed diseases and prevent them, whether for prophylactic or metaphylactic purposes, the administration of classes of antibiotics common to those used in human medicine is one of the biggest concerns related to the farming industry [20]. In addition, the use of broad-spectrum antibiotics in animals has a greater potential to cause the development of extensive resistance [21].

Critically important antimicrobials (CIAs) include antibiotics that are used for the treatment of serious bacterial infections in humans, in cases where they represent the only therapeutic option or when available alternative therapies are limited. In addition, this classification also includes therapies used to treat bacterial infections where there is a risk of acquiring resistance [20]. The highest priority of CIAs includes quinolones, cephalosporins of the third generation or more, macrolides, ketolides, glycopeptides and polymyxins [22]. An example of a polymyxin is colistin, which is considered an antimicrobial of last resort against multidrug-resistant Gram-negative bacteria. Nonetheless, resistance to colistin has emerged due to horizontal transfer mediated by plasmids [23].

Occasionally, antibiotics are improperly added to feed as growth promoters, which is a major concern and an illegal practice because it causes selective pressure on bacteria and favours the spread of resistant bacterial strains, which can consequently be transmitted to other animals and humans [22,24]. However, there is also substantial concern about the manufacturing process of medicated feed which can lead to cross-contamination. In this context, it is crucial to properly clean the equipment used in the production of medicated feed to avoid cross-contamination to other non-medicated feeds. In this way, the unintentional administration of these substances to farm animals can be minimised [13,25].

### 1.1. The History of Growth Promoters in Animal Production

In 1940, the use of *Streptomyces aureofaciens* was introduced as a probiotic to promote weight gain in animals, which resulted in the discovery of chlortetracycline [26]. Chlortetracycline demonstrated the ability to increase the growth rate of chicks, which subsequently led to the expansion of its use to other animals, such as pigs and cattle [17,27]. In 1951, the Food and Drug Administration (FDA) of the United States of America approved the use of antimicrobials as food additives without the need for a veterinary prescription [28]. In the 1970s, studies showed that the use of penicillin as a growth promoter resulted in a weight increase of approximately 11%, while tetracyclines provided an increase between 8 and 10% [26]. However, over the years, these antibiotics have shown the highest rates of resistance [20].

Some benefits attributed to antimicrobial growth promoters have been suggested by scientific studies, such as modulation of the intestinal microbiota, which results in greater efficiency in nutrient utilisation due to a reduction in microbial competition. Likewise, they can favour better nutrient absorption due to the reduction in the thickness of the intestinal epithelium, and contribute to the prevention of pathogens, as well as reducing bacterial toxins that can affect growth. As a result, antimicrobial growth promoters favour feed efficiency, promote weight gain and reduce feed consumption [28,29,30].

Antimicrobial growth promoters have traditionally been administered through medicated feeding in the intensive system, especially in the USA [31]. However, due to the alarming increase in antimicrobial resistance, policies restricting their use have since been implemented. It is estimated that infections caused by antibiotic-resistant bacteria result in approximately 700,000 human deaths annually worldwide, and if the AMR crisis is not properly controlled, it could cause more than 10 million human deaths by 2050 [32]. Several measures have been implemented to solve this problem. As far as farmers are concerned, the “Yellow Card” policy has been implemented since 2016, which penalises those who fail to meet the targets for reducing the use of antimicrobials [18,22]. In addition, the administration of antibiotics for the prevention of infectious diseases and antibiotics as growth promoters has been restricted [20]. Other measures include imposing restrictions on veterinarians, preventing them from making a profit from antimicrobial prescriptions, banning the use of CIAs and carrying out audits in the veterinary field [21,22].

**Figure 1:**
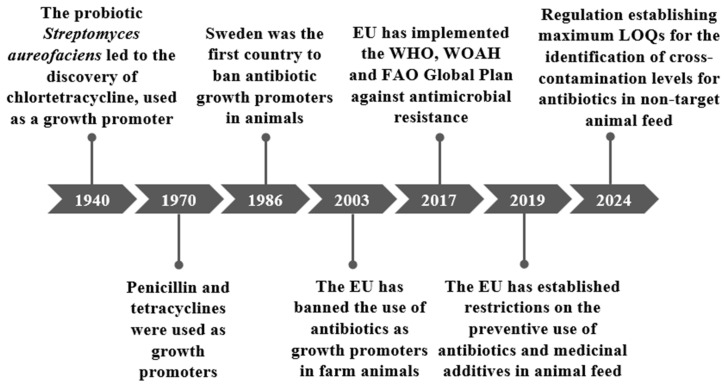
Key historical events in the regulation of growth promoters.

In 1986, Sweden became the first country to ban the use of antibiotic growth promoters in animals (Figure 1). Subsequently, the European Commission implemented Regulation (EC) No 1831/2003, which came into force in 2006, establishing a ban on the use of these antibiotics as growth promoters [30,33].

In 2015, the World Health Organisation (WHO), the World Organisation for Animal Health (WOAH) and the Food and Agricultural Organisation of the United Nations (FAO) produced a Global Action Plan on AMR. This plan is based on the One Health approach, which recognises the correlation between environment, animal health and human health [18]. In 2017, the European Union (EU) adopted and implemented this plan, which reinforced its commitment to combating antimicrobial resistance. In this context, the European Commission emphasised the need for greater investment in research, surveillance systems and strengthening the regulatory framework for veterinary medicines and medicated foods [22]. Within the framework of European Union legislation, Regulation (EU) 2019/6 on veterinary medicinal products and Regulation (EU) 2019/4 on medicated feed-imposed restrictions on the use of antibiotics in livestock farming for preventive purposes and feed additives [23,34,35].

Due to the regulatory restrictions imposed on antimicrobial growth promoters in order to control and prevent the development of AMR, there was initially a negative impact on the health of farm animals. This ban resulted in an increase in the use of antimicrobials to treat infectious diseases. As an alternative, ionophores have been administered as food additives, as they have antimicrobial activity against Gram-positive bacteria and protozoa and are not used in human medicine due to their toxicity [36,37,38,39]. Ionophores are widely used in the USA to prevent the disease coccidiosis, a disease that causes weight loss in animals, and are therefore also used as growth promoters. In the European Union, Article 11 of Regulation (EC) No 1831/2003 allowed their use as additives with a coccidiostatic function until 2012. After that, restrictions were imposed, and their use was limited to therapeutic purposes only on veterinary prescription [29,36,40,41].

#### Alternative Growth Promoters and Disease Control

As a result of all the restrictions described above, research has intensified into the development of safer alternative growth promoters, such as phytochemicals, prebiotics, exogenous enzymes, antimicrobial peptides, probiotics and medicinal plant extracts. These alternatives have shown positive effects on feed digestibility and body weight gain in various animals such as sheep, chickens, turkeys, piglets, goats and cattle [42,43,44,45]. Alternatives are being adopted which, as well as not posing a risk to public health, provide benefits in terms of increasing growth rates and, above all, protecting the host against pathogenic bacteria. These include antimicrobial peptides, substances that can be produced by various sources, including bacteria, which are called bacteriocins, as well as fungi, plants and invertebrates, particularly insects that synthesise defensins. These compounds are considered food additives that protect the host, are recognised as safe and their environmental impact is reduced [16,46]. Other strategies are predatory bacteria that fight pathogens, bacteriophages and increased vaccination to boost immunity [16,32,33].

### 1.2. Environmental Impacts of Livestock

The lack of proper cleaning in factories that produce medicated feed and also manufacture non-medicated feed, or during transport, enables cross-contamination, which can result in the unexpected presence of residues in animal products. The insertion of these residues into the food chain can pose a risk to public health, since human exposure to such substances can cause adverse effects such as muscle tremors, nausea, dizziness and muscle pain [47]. In this context, with a view to protecting human health and the strict control of these residues, the European Regulation (EU) No 37/2010 establishes the maximum residue limits (MRLs) of pharmacological substances admissible in products of animal origin, in accordance with the principles established by the Codex Alimentarius Commission [1,48,49].

As the urban population grows, livestock companies locate themselves close to cities to facilitate supply, but this proximity increases the risk of transmitting zoonotic diseases [19]. As a result, the management of waste resulting from the livestock industry is one of the main environmental concerns, as it can compromise the quality of water and soil, which enhances the transmission of zoonotic microorganisms and antibiotic pollution [50].

Antibiotic pollution refers to the excretion of antibiotic residues, not absorbed by animals, into the environment [51]. In more serious situations, the presence of these residues in food products or the environment can cause adverse effects in humans. Various effects in humans have been associated with certain antibiotics, for example, the penicillin, aminoglycosides and tetracyclines are linked to hypersensitivity reactions. Gentamycin can cause nephropathy. Chloramphenicol has been associated with bone marrow toxicity. Sulphamethazine, oxytetracycline and furazolidone have carcinogenic potential and streptomycin can cause loss of balance [12]. These residues are considered pollutants, as they can be toxic and negatively affect the microbiota of soil and water, which can consequently lead to the emergence of resistant bacteria [11,51].

**Figure 2:**
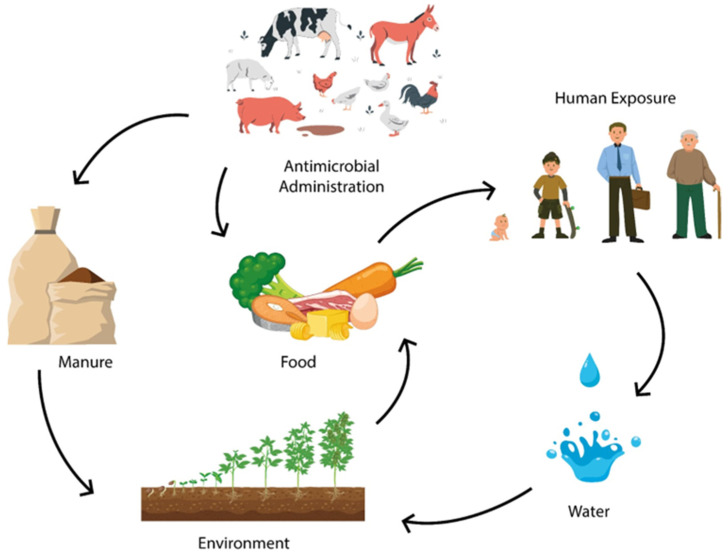
The use of antibiotics as growth promoters in animal production contributes to antimicrobial resistance in the environment, water, and manure, reaching humans through food, posing a challenge within “One Health” perspective.

This situation poses a risk to human and animal health, either directly or indirectly, through contact with the water and food produced (Figure 2) [50].

Despite the benefits associated with increasing weight and reducing costs in food production, growth promoters have significant negative effects. Antimicrobial growth promoters are generally used in low concentrations, which are insufficient to completely eliminate pathogens, but sufficient to encourage the selection of resistant bacteria [23,52]. Transposons, plasmids, and other mobile genetic elements may contain resistance genes that can be transmitted between bacteria by horizontal transfer. This selection of resistant bacteria is not only limited to pathogenic bacteria but also to commensals. Resistance can be transmitted by horizontal transfer or developed indirectly through antibiotic residues present in water, soil and contaminated foods such as milk, eggs and meat [1,53].

The greatest risk lies in the transfer of resistance genes between pathogenic bacteria and the host’s intestinal microbiota, representing a mechanism for spreading AMR with public health implications [16]. Resistant bacteria such as *Escherichia coli* and bacteria from the *Enterococcus* genus are present in farm animals and have the ability to transfer resistance genes to bacteria of the *E. coli* species and the *Enterococcus* genus from the intestinal flora of humans or other animals, which contributes to an increase in resistance to multiple antimicrobials [17].

### 1.3. Review on Analytical Methodologies for Analysis of Pharmaceutical Residues in Feed

In recent years, microbiological and enzyme-linked immunosorbent assays (ELISA), have been widely used to detect the presence of antibiotics in feed. For example, like the fluoroimmunoassay, it uses antibodies to detect the analyte and fluorescent markers to generate a signal. These methods stand out because they allow quick analyses, are easy to perform by non-specialist technicians and can be carried out in tubes or microplates [11,12]. At the same time, there has been an increase in the use of biosensors for antibiotic detection, mainly due to the speed of analysis, the possibility of automation and use at the sampling site. However, there are limitations, namely the potential instability of the sensing biological element [11]. Although these methods are suitable for sample screening, they do not guarantee precise quantification or analytical stability [25,54].

Physicochemical instrumental methods of analysis for screening and confirmatory purposes are currently being used, as they are more specific and selective. However, they have disadvantages, such as high cost, long execution time and the need for qualified professionals [11]. Commonly, chromatographic separation is achieved through interactions between two phases in a stationary phase, a chromatographic column, and the mobile phase, usually composed by a mixture of solvents, which transports the analytes along the column. Gas chromatography (GC) is used to separate volatile compounds, while liquid chromatography (LC) is suitable for analysing non-volatile and thermally labile substances, which makes it useful in pharmaceutical analyses. In liquid chromatography, the separation of analytes results from the different affinities they establish with the stationary and mobile phases. Chemical species that establish strong interactions with the stationary phase exhibit a decreased elution rate, resulting in longer retention times. In contrast, analytes that interact weakly with the stationary phase have a shorter retention time [48,55].

High-performance liquid chromatography (HPLC) is the most widely used method for detecting veterinary medicines in animal feed due to its high precision. HPLC makes it possible to control various parameters more precisely in order to optimise separation efficiency and resolution. The composition of the mobile phase, in terms of the type of solvents or the gradient profile, influences the retention times of the analytes. The temperature of the column affects the interaction between the analytes and the stationary phase. The flow rate regulates the speed of the analytes, so high flow rates reduce the analysis time, but can compromise resolution. Proper control of these parameters allows effective separations to be achieved, even in complex mixtures [25,55].

With the advancement of analytical techniques, the ultra-high-performance liquid chromatography (UHPLC) system has been widely used, as it allows for a reduction in analysis time, especially in routine analyses, as well as reducing the consumption of solvent volume, contributing to the mitigation of environmental impacts. In addition, it provides greater resolution and sensitivity [56,57]. This technique can be complemented with various detection methods, such as fluorescence or ultraviolet absorption, but they do not provide structural information and have limitations for the simultaneous detection of analytes. On the other hand, the use of a mass spectrometry (MS) detector enables the determination of polar organic compounds at low concentrations, in the µg/kg and ng/kg range, when associated with UHPLC, more specifically UHPLC-MS. The UHPLC system provides an increase in yield and resolution, while advances in mass spectrometry contribute to increased sensitivity and selectivity [48,54,58]. Ultra-high-performance liquid chromatography coupled with tandem mass spectrometry (UHPLC-MS/MS) has been widely used for the detection of antibiotics in animal feed [59]. In mass spectrometry, a technique based on measuring the mass-to-charge ratio (*m*/*z*) of ions, the triple quadrupole analyser is often used as the predominant instrument for the targeted analysis of compounds. From another perspective, ultra-high-performance liquid chromatography coupled to a time-of-flight detector (UHPLC-ToF-MS) is a system often used for non-targeted analyses [58]. This system is mainly used for the quantification of antibiotics in complex biological matrices, such as the liver and kidney of animals, and has potential for use in feed. This detection system is promising for screening, since it allows precise mass identification and the ability to multi-detect an unlimited number of compounds in a single run [56,60,61].

Several recent studies have been proposing HPLC-MS/MS-based methods for the detection of antibiotics in animal feed. However, differences in the classes of antibiotics analysed and in the sample preparation and extraction protocols make it difficult to compare the reported results. Therefore, this review aims to highlight the main methodologies used for the detection of medicine veterinary medicines in feed intended for food-producing animals, as well as to identify topics that remain underexplored in the research.

## 2. Methods

As part of this review, a research question was developed that encompassed validated analytical methods for determining antibiotics in animal feed. To this end, a qualitative thematic analysis was chosen. A bibliographic search was conducted using scientific databases such as PubMed, ScienceDirect, Scopus, Google Scholar and European Union regulations.

Boolean operators “AND” and “OR” were used to combine search terms, such as “Antibiotics”, “Veterinary residues”, “Animal feed”, “Liquid chromatography”, “Mass spectrometry”, were used to converge the scope of the results. Articles were selected according to the following criteria: articles in English, articles published in the last 5 years, articles whose scope is related to extraction/purification procedures and methods for the analysis of veterinary drugs. The work focused mainly on antibiotics, due to the problem of antimicrobial resistance.

The scope of the target matrices was expanded to include feeds of different formulations such as premixes, compounds, concentrates, supplements and additives, and also for different species such as pigs, cattle, poultry, rabbits, fish, shrimps, crabs and horses.

## 3. Results and Discussion

In the studies referred to in Table 1, studies such as [54,62,63,64,65,66,67,68,69,70,71,72,73], the validation of detection methods was carried out on various types of feed and/or for different types of animals, which allows for a closer approximation to routine reality, considering that feeds have a wide diversity and complex composition. For animals such as swine, poultry, cattle, sheep, and rabbits, feed composition may vary between barley, linseed, corn, alfalfa, sunflower cake, bone meal, horse bean, bean pulp, triticale, rapeseed, palm oil, sugar beet, soybean, fish meal, wheat, and wheat bran [66,69]. On the other hand, fish feed has a higher lipid, protein, and moisture content [63,74]. Regarding wet matrices in the study by [68], they evaluated the moisture content and found that feed with 40% moisture had stable recovery results, which agrees with the study by [75]. These complex matrices can cause signal suppression or amplification, which affects the response of the analytes. Hence, the importance of a good extraction and clean-up procedure to prevent matrix effects and equipment deterioration.

In the following sessions, a detailed presentation of the most representative methods available in the literature is presented. Those methods, from the standard solutions preparation and extraction procedure are summarised in Table 1.

Table 2 shows the relationship between the chromatographic conditions and the results of the performance parameters.

### 3.1. Standard and Sample Preparation

Depending on the solubility characteristics of the compounds analyzed, the appropriate solvent should be used for the preparation of the needed standard solutions. This step is important to guarantee the stability of the solutions used for the accurate detection and quantification of the target compounds.

Liquid chromatography coupled with tandem mass spectrometry (LC-MS/MS) is the most often used detection technique, for greater robustness, in the simultaneous analysis of multi-class medicines in animal feed. Before this analysis, feed samples should undergo a preparation and extraction procedure. The feed sample preparation comprises a grinding step. Together with the unknown sample analysis, a calibration curve may be prepared by spiking blank samples with the antibiotics and internal standards, shaken and kept in equilibrium with the feed matrix, at room temperature, and preferably protected from light. It has been found that the most used standard solution solvents are methanol, acetonitrile, ultrapure water, or mixtures of these solvents. The use of the internal standard proved to be essential in order to correct for possible detection fluctuations and the constant effects in the feed matrix.

### 3.2. Extraction/Clean-Up Procedures

Several authors have used extraction and purification steps such as SPE, d-SPE, LLE, QuEChERS, protein precipitation through freezing and dilution prior to analysis. Ideally, the best extraction and clean-up procedure should be combined for multi-detection of target compounds most commonly used in veterinary medicine.

In the study [76], for amoxicillin, an antibiotic widely used in veterinary medicine, the extraction solvent that had the best extraction recovery was the 0.01 M potassium dihydrogen phosphate (KH_2_PO_4_) buffer solution (pH 4.5). In the study by [77], 250 μL disodium salt ethylenediaminetetraacetic acid (Na_2_EDTA) and 6 mL acetonitrile (ACN), a different solvent, were used as they validated it for amoxicillin and other compounds that have a different chemical composition, such as chlortetracycline, doxycycline, lincomycin, oxytetracycline, sulfadiazine, sulfadimethoxine, tiamulin, tilmicosin, trimethoprim, and tylosin. In comparison, the procedure by [77] includes more steps such as evaporation under nitrogen flow and LLE for lipid removal. Na_2_EDTA was used to improve the extraction of tetracyclines, as these antibiotics bind to metal ions. Similar studies, such as [64,71,78], took the same care to add ethylenediaminetetraacetic acid (EDTA).

The studies by [71,78] present similarities in the extraction solution, using 0.04% formic acid (FA) in Milli-Q water/0.1% FA in methanol (MeOH) (90/10) and 0.1% FA in water/ACN, (98/2), respectively, but the procedure in [71] stands out for using freezing to precipitate impurities, while [78] uses evaporation under nitrogen flow, which is in line with [64] and [77]. To purify tetracyclines, the study [64] used a solid-phase extraction (SPE) step with Strata-X-CW cartridges, as it had better efficiency compared to C18, HLB and Strata-X cartridges.

In the study by [54], most target compounds such as tylosin, tiamulin, trimethoprim, and sulfadiazine are also detected by the study [77], but they performed a simpler cleaning procedure with dilution and filtration. The recent study by [65] also detects tylosin and other antibiotics such as tylvalosin and tilmicosin, which are weakly alkaline and lipophilic macrolides. To separate the antibiotics from the interferents, they performed two centrifugations and used magnetic separation with 30 mg of Magnetic Mixed-mode Cation eXchange (M-MCX) beads.

In the study [72], they validated a method for the detection of trimethoprim, diaveridine, and ormetoprim, substances widely used to treat coccidiosis in veterinary medicine. To remove lipids, they performed LEE twice with n-hexane for efficient purification. Compared to chloroform, dichloromethane together with sodium acetate achieved greater selectivity.

The aminoglycoside class of antibiotics is used in both veterinary and human medicine. They are polar, water-soluble, polybasic cations, as their structure contains hydroxyl and amine groups and they can be present in polyionic forms. According to the study by [66], for aminoglycoside antibiotics (apramycin, paromomycin, tobramycin, neomycin) and aminocyclitol (spectinomycin) to be extracted, it is best to use a strongly acidic extraction solvent with ionic strength such as 10 mM ammonium acetate, 0.4 mM EDTA, 0.5% sodium chloride (NaCl) and 2% trichloroacetic acid (TCA), in order to precipitate interferents and prevent the target compounds from binding with impurities. Similarly, in the validation for aminoglycosides (paromomycin, amikacin, tobramycin, gentamycin, streptomycin, kanamycin, hygromycin B, apramycin, dihydrostreptomycin) and aminocyclitol (spectinomycin), the study by [63] used 10% TCA water solution containing 2 mmol/L of Na_2_EDTA as the extraction solvent, since after testing with various percentages of TCA, they found that 10% TCA obtained better recoveries in aquaculture feed. In the cleaning stage, 70% ACN was used to precipitate impurities, followed by SPE with C18 adsorbent by direct passage of the analytes, as these have no affinity with C18. This adsorbent was more efficient compared to HLB, weak cation exchange (WCX), and mixed-mode cation (MCX). On the other hand, the aforementioned study by [66] selected the HLB adsorbent for purification and found that pH 4 is ideal for retaining analytes, preventing losses due to coprecipitation.

The study by [70] validated a method for cyclopolypeptide antibiotics, in which they used two extraction solutions with the same type of solvents but varying proportions, first MeOH/2% FA aqueous solution (2:5, *v*/*v*) and then (5:2, *v*/*v*), due to differences in polarity and solubility between the analytes. For the purification stage, they used d-SPE with the primary secondary amine (PSA) adsorbent, as it not only separates interferents but also chelates metal ions.

In the study by [62], they also validated a method for colistin/polymyxin E, bacitracin, virginiamycin M1, in which they used a different extraction solvent, ACN/10% TCA (60/40, *v*/*v*), however it remains an acid extraction to reduce the strong binding of colistin to proteins. For cleaning, they performed SPE with HBL, testing SPE in classic retentive mode and pass-through mode, but the most effective was the retentive mode with HLB. In addition, the study of [67] validated a method for the detection of bacitracins, in which they used a basic extraction solvent, ACN/MeOH/15% ammonia (1:1:1), which differs from the studies [62,70]. As this solvent has an organic and an aqueous phase, it easily extracts bacitracin, as it is soluble in water and methanol. The ammonia solution is useful for extracting bacitracin zinc and bacitracin methylene disalicylate, which have different solubility from bacitracin. In the purification stage, SPE with HLB was rejected due to the strong retention of bacitracin and bacitracin zinc, which did not allow complete elution. SPE with C18, on the other hand, had good recovery rates for bacitracin and bacitracin methylene disalicylate, but bacitracin zinc was strongly retained and prevented elution. However, with the addition of EDTA solution before SPE, excellent recovery rates were obtained for all bacitracins.

Several methods have been validated for the multi-detection of veterinary drugs, including in various classes of antibiotics due to the major problem of antimicrobial resistance.

In 2010, in the study by [79], they validated the method for 13 classes of antibiotics, using MeOH/ACN/McIlvaine, pH 4.6 (37.5/37.5/25, *v*/*v*) with 0.3% Na_2_EDTA as the extraction solution. When they tested only organic solvents, they did not obtain good results for the penicillin, tetracycline and macrolide families. In the case of tetracyclines, they observed that when using solutions with formic acid or solutions with EDTA, they obtained similar results. SPE steps (C18 and HLB) were also compared, but good recoveries were not obtained, so they opted for the use of d-SPE with PSA for the removal of carbohydrates and fatty acids based on the QuEChERS method. They concluded that the addition of 1% formic acid did not allow good results for ionophores, so the ideal pH is around 4.5 for the target compounds to bind to PSA. Similarly, in a 2020 study [69], they used ACN/water/FA (79/20/1, *v*/*v*/*v*) as the extraction solution and found that the QuEChERS method with PSA and 1% formic acid had greater matrix effects when compared to a simple dilution step (1:1).

Furthermore, in the study by [68], they used MeOH acidified with 1% acetic acid as the extraction solvent, as they obtained better recoveries of bacitracin and minocycline compared to MeOH with 1% formic acid. For the purification step, they used the Quick Polar Pesticides (QuPPe) method modified in d-SPE with C18 for multiple analysis of polar or semipolar veterinary drugs. However, in the study by [73], they also used MeOH as the extraction solvent, but without acidification. They tested MeOH and ACN with and without acid and observed that MeOH obtained better results for protein precipitation and superior extraction of analytes. They found that after evaporation under nitrogen flow and reconstitution with ACN/water (30/70, *v*/*v*), low matrix effects were obtained.

In contrast, the study by [75] used ACN as the extraction solvent. However, in the pre-treatment, they added 0.25 M EDTA-Mcilvaine buffer (pH 4.0), as tetracyclines and fluoroquinolones are sensitive to pH and tend to chelate. To complete the extraction, salt was added based on the QuEChERS method, followed by centrifugation and filtration.

On the other hand, the [74] method used ACN/MeOH (75/25, *v*/*v*) solution for extraction, as it yielded better recoveries compared to the same solution with acetic acid and ACN solution. Similarly, they applied the QuEChERS method, i.e., the use of salts and d-SPE with Zsep+ adsorbent. They then proceeded with evaporation under nitrogen flow, reconstitution, and filtration.

In the study by [80], the extraction solvent was ACN/water (80/20, *v*/*v*), and this acid-free solution showed higher recoveries. They proceeded with centrifugation, followed by dilution with ACN/water (20/80, *v*/*v*) and finally filtration, as other purification procedures such as the QuEChERS method, QuEChERS with PSA or C18 and freezing did not yield better results. For this reason, dilution remains the preferred method because it is simple and quick for routine analyses, provided that the dilution is (1:1) so as not to compromise sensitivity.

**Table 1:** Detailed description of standard solution solvents, extraction/clean-up procedures for the determination of veterinary drugs in different feed matrices.

Ref.	Feed Matrix/Weight	Analyte	Working Solution	Extraction Solvent	Extraction/Clean Up Method
[76]	Premixes	Amoxicillin	LC water	25 mL of a 0.01 M ${KH}_{2}{PO}_{4}$ solution (pH 4.5)	Rotary mixer (30 min), centrifugation (3400× *g* 10 min), filtration (0.2 μm), dilution 1:100
[79]	Bovine, lamb, piglet feed (4 g)	33 analytes (tetracyclines, quinolones, penicillins, ionophore coccidiostats, macrolides, sulfonamides, quinoxalines, phenicols, lincosamides, diaminopyrimidines, polypep tides, streptogramins and pleuromutilins)	No information	15 mL of MeOH/ACN/McIlvaine, pH 4.6 (37.5/37.5/25, *v*/*v*) with 0.3% ${Na}_{2}$EDTA	Agitation (30 s), ultrasonic bath for 15 min (37 kHz, 300 W), centrifugation (4500× *g* 10 min 15 °C), d-SPE (250 mg PSA), evaporation under nitrogen flow (40 °C), dilution 1:1.5 with ultrapure water, storage −20 °C, centrifugation (5000× *g*, 10 °C), dilution 1:4 with water
[73]	Pig, cattle, poultry feed (5 g)	50 analytes (aminocoumarin, amphenicols, beta-lactams, lincosamide, macrolides, diaminopyr imidine, quinolones, sulfonamides, streptogramin, pleuromutilin, polypeptide, quinoxaline, tetracyclines, benzimidazoles	MeOH	25 mL MeOH	Agitation (15 min), centrifugation (4650× *g*, 10 min), organic phase evaporated to dryness under nitrogen flow (50 °C), reconstitution with ACN/water (30/70, *v*/*v*), centrifugation (11,500× *g*)
[72]	Complete (laying hens) (2 g), concentrated (pigs) (1 g), premix (laying hens) (1 g)	Trimethoprim, diaveridine, ormetoprim	MeOH/0.2% formic acid in water (20:80, *v*/*v*)	10 mL sodium acetate and 20 mL dichloromethane	Ultrasonic bath (30 min), centrifugation (1800× *g*, 15 min), extract centrifugation (1800× *g*, 10 min), evaporation under nitrogen flow (40 °C), reconstituted in MeOH/0.2% FA in water (20/80, *v*/*v*), LEE (2 times) with n-hexane, centrifugation (1800× *g*, 10 min), filtration (0.22 μm)
[78]	Milk farm feed (2 g)	Tetracycline, chlortetracycline, doxycycline, oxytetracycline	MeOH	300 μL TCA, 8 mL McIlvaine buffer/EDTA and 6 mL ethyl acetate	Orbital shaker (200 rpm, 20 min), centrifugation (4500 rpm, 15 min), supernatant evaporated to dryness, dissolved with 0.04% FA in Milli-Q water/0.1% FA in MeOH (90/10), vortexed, centrifugal filter (9000 rpm, 10 min)
[64]	Swine, poultry feed (5 g)	Oxytetracycline, tetracycline, chlortetracycline, doxycycline	MeOH	25 mL McIlvaine/${Na}_{2}$EDTA buffer (pH 4)	Agitation (45 min), centrifugation (4000 rpm, 20 min, 20 °C), SPE (Strata-X-CW—elution with 2% FA in MeOH), evaporation under nitrogen flow (40 °C), resuspended 1 mL 0.1% FA in deionised water
[71]	Premixes, supplements, concentrates, feed (poultry, swine, bovine, equine, prawns) (1 g)	24 analytes (fluoroquinolones, sulfonamides, tetracyclines, trimethoprim)	MeOH	3 mL MeOH/0.1% FA in water, 70/30)	Orbital shaker (20 min), centrifugation (4000× *g*, 10 min, 5 °C), freezing (30 min, −18 °C), centrifuged supernatant 12,000 g (20 min, 5 °C), addition of 1 mL 0.1% FA in water/ACN, (98/2)
[54]	Swine, poultry and cattle feed (2 g)	Tiamulin, trimethoprim, tylosin, sulfadiazine, sulfamethazine	MeOH and ACN for sulfadiazine	10 mL of 0.1% FA in ACN	Orbital shaker (30 min), centrifugation (7000 rpm, 10 min, 20 °C), dilution (1:19) with Milli-Q water, vortex, filtered
[70]	Premix (piglet, pig), complete (poultry, pig), feed additive (pig) (1 g)	Cyclopolypeptide (vancomycin, polymyxin B, polymyxin E/colistin, teicoplanin A2, bacitracin A, daptomycin) and virginiamycin M1	MeOH/0.1% FA in water (50/50, *v*/*v*)	1. MeOH/2% FA aqueous solution (5 mL) (2:5, *v*/*v*) 2. MeOH/2% FA aqueous solution (5 mL) (5:2, *v*/*v*)	Solution 1, agitation (20 min), centrifugation, re-extraction with solution 2, combined supernatants, d-SPE (50 mg PSA), centrifugation (9000 rpm, 5 min), filtration (0.22 μm)
[63]	Fish, prawn, crab feed (2 g)	Aminoglycosides (paromomycin, tobramycin, gentamycin, kanamycin, hygromycin B, apramycin, streptomycin, dihydrostreptomycin, amikacin) and spectinomycin	Water	6 mL of 10% TCA water solution containing 2 mmol/L of ${Na}_{2}$EDTA	Agitation (2 min), ultrasonication (10 min), centrifugation (10,000 rpm, 10 min), 70% ACN to purify the extract (7:3) in SPE (C18), vortex, filtration (0.22 μm)
[77]	Pigs, poultry, rabbits feed (1 g)	Amoxicillin, chlortetracycline, doxycycline, lincomycin, oxytetracycline, sulfadiazine, sulfadimethoxine, tiamulin, tilmicosin, trimethoprim, tylosin	Ultrapure water	250 μ$L\;{Na}_{2}$EDTA and 6 mL ACN	Agitation (10 min), centrifugation (20,000× *g*, 5 min, 4 °C), evaporation under nitrogen flow (50 °C), ammonium acetate recovery, vortex, LEE with isooctane, filtration (0.45 μm)
[69]	Compound (cattle, chicken) (5 g)	1200 analytes (162 veterinary drug)	ACN/water/FA (49.5/49.5/1, *v*/*v*/*v*)	20 mL ACN/water/FA (79/20/1, *v*/*v*/*v*)	Rotary shaker (90 min), dilution (1:1) of extracts with ACN/water/FA (79/20/1, *v*/*v*/*v*)
[62]	Pigs, poultry, rabbits feed (1 g)	Colistin/polymyxin E, bacitracin, virginiamycin M1	0.5% FA in water	8 mL ACN/10% TCA (60/40, *v*/*v*)	Rotary shaker (100 rpm, 10 min), centrifugation (20,000× *g*, 10 min, 4 °C), supernatant dilution with water (1:1), SPE (HBL-elution with 0.2% FA in MeOH), evaporation under nitrogen flow (45 °C), recovery 0.5% FA in water/MeOH (90/10, *v*/*v*), vortex, centrifugation (20,000× *g*, 5 min, 4 °C)
[75]	Compound (livestock, pets) (5 g)	258 analytes (56 veterinary drugs)	No information	10 mL ACN	Rotary shaker (3000 g, 1 min), modification of the QuEChERS: 4 g of MgSO_4_, 1 g of NaCl, 1 g of Na_3_C_6_H_5_O_7_, 0.5 g of Na_2_C_6_H_6_O_7_, shaking (3000× *g*, 1 min), centrifugation (3000× *g*, 10 min), syringe filter (0.2 μm) with 0.25 M (EDTA)-Mcilvaine buffer (pH 4)/ACN/sample (60/30/10, *v*/*v*/*v*)
[68]	Corn, cow, pet feed (2.5 g)	30 veterinary drugs/17 classes (penicillins, quinolones, cephalosporins, sulfonamides, tetracycline, amphenicols, coccidiostats, polypeptides, lincosamides, macrolide, nitroimidazole, anthelmintics, phenylhydrazines, pyrethrins, neuroleptic agents, triazene trypanocidal agents)	ACN	1% acetic acid in MeOH	Shaking (1500 rpm, 10 min), centrifugation (3000 rpm, 10 min), modification of the QuPPe (d-SPE with 100 mg C18), shaking (1500 rpm, 1 min), centrifugation (3000 rpm, 10 min), syringe filter (0.2 μm)
[80]	Poultry feed (0.5 g)	144 compounds/15 classes (tetracyclines aminogylcosides, penicilines, macrolides, amphenicols, beta-lactams, cephalosporines, quinolones, polymyxin, polypeptide, glycopeptides, dihydrofolate reductase inhibitors, nitroimidazole, pleuromutilines, polyether ionophores)	600 μL multianalyte mix with 400 μL ACN/water (50/50, *v*/*v*)	2 mL ACN/water (80/20, *v*/*v*)	Rotary shaker (90 min), centrifugation (3500 rpm, 10 min), dilution (1:1) extract with ACN/water (20/80, *v*/*v*), filtration (0.2 μm)
[66]	Compound (poultry, swine, bovine, ovine, rabbit) (2 g)	Aminoglycoside (apramycin, paromomycin, tobramycin, neomycin) and spectinomycin	ACN/water/acetic acid (20/78/2 *v*/*v*/*v*)	20 mL of the 10 mM ammonium acetate, 0.4 mM EDTA, 0.5% NaCl and 2% TCA	Wrist shaker (30 min), centrifugation (3200× *g*, 10 min, 4 °C), pH adjustment to 4 with KOH, centrifugation (3200× *g*, 10 min, 4 °C), SPE (500 mg of HLB—elution with 3 mL 10% *v*/*v* FA, 5% *v*/*v* isopropanol in water)
[74]	Fish feed	24 pharmaceuticals, among them 5 antibiotics (sulfadiazine, sulfamethazine, sulfamethoxazole, sulfapyridine, trimethoprim)	MeOH	10 mL ACN/MeOH (75/25, *v*/*v*).	QuEChERS: addition of 6 g MgSO_4_ and 1.5 g sodium acetate, d-SPE (50 mg Zsep+), vortex, evaporation under nitrogen flow, reconstitution with 0.1% FA in water/methanol (90/10, *v*/*v*), filtration (0.22 μm)
[67]	Concentrated (chicken, cow, swine), formulated (chicken, fish, swine), premixed (chicken, cow, duck, fish, swine), concentrate supplement (cow) (2 g)	Bacitracin, bacitracin zinc and bacitracin methylene disalicylate	No information	10 mL ACN/MeOH/15% ammonia (1:1:1)	Shaking (15 min), 2 centrifugations (9500 rpm, 5 min), dilution (1:2) with EDTA solution (1.5 mmol/L, pH 7.0), SPE (C18—elution with 15% ACN solution, evaporation under nitrogen flow (40 °C), resuspension in 20% MeOH solution, filtration (0.22 μm)
[65]	Additive premixes, concentrate supplements, compound, concentrated feeds (2 g)	Macrolide (tylosin A, tylvalosin, tilmicosin)	5% ammoniated MeOH	10 mL of 50% ACN	Vortex shaking (300 rpm, 15 min), centrifugation (7000 rpm, 5 min), re-extraction with 10 mL of 50% ACN, combining and homogenising the two supernatants, magnetic separation with 30 mg of magnetic M-MCX beads—elution with 5% ammoniated MeOH), filtration

**Table 2:** MS (/MS) detection conditions from the studies discussed regarding the analysis of veterinary drugs in animal feed.

Ref.	Type/Ion. Mode/Op. Mode	Column/T	Mobile Phases	Flow Rate/Total Run Time	Recovery	LOD/LOQ/ (µg/kg)	RSD_r_/RSD_R_
[76]	LC-MS/MS/ESI+/MRM	PLRP-S polymeric column (150 mm × 2.1 mm i.d., 100 Å par ticle size: 3 m) + pre-column of the same type (5 mm × 3.0 mm i.d.)/room temperature	(A)—0.1% FA in water (B)—ACN	0.2 mL·min^−1^/18 min	No information	1030/3400	≤15%
[79]	HPLC-MS/MS/ESI− and ESI+/MRM	Zorbax XDB plus (2.1 mm × 150 mm, particle size 3.5 m; Agilent, Germany), + guard column (2.1 mm × 12.5 mm, 5 μm)/40 °C	(A)—(ESI-) water with 5 mM ammonium acetate, (ESI+) 0.1% FA in water (B)—(ESI-) ACN/MeOH (70/30, *v*/*v*); (ESI+) ACN/MeOH (70/30, *v*/*v*) with 0.1% FA	0.25 mL·min^−1^/28 min	51–116%	No information/3.8–65	≤15.4%/≤18.6%
[73]	UHPLC-MS/MS/ESI− and ESI+/MRM	Acquity UPLC HSS T3 column (150 mm × 2.1 mm, 1.7 μm)/50 °C	(A)—(ESI-) water, (ESI+) 0.05% FA in water (B)—(ESI-) ACN, (ESI+) 0.05% FA in ACN	0.5 mL·min^−1^/9 min	No information	No information	No information
[72]	HPLC-MS/MS/ESI+/MRM	Agilent Eclipse Plus C18 column (3.0 mm 100 mm, 1.8 μm) + prefilter (4 mm and 5 μm)/40 °C	MeOH/0.2% FA (20/80, *v*/*v*)	0.3 mL·min^−1^/no information	74.4–105.2%	20/40	<7.4%
[78]	HPLC-MS/MS/ESI+/MRM	Sunfire C18 column (150 mm × 2.1 mm i.d., 5.0 mm)/35 °C	(A)—Milli-Q water with 0.04% FA (B)—MeOH with 0.1% FA	0.25 mL·min^−1^	78–111%	24–100/40–150	<17%/<23%
[64]	LC-MS/ESI+/SIM	Kinetex C18 analytical column (100 mm × 4.6 mm, 2.6 µm) + C18 guard cartridge/20 °C	(A)—0.1% FA in water (B)—0.1% FA in ACN	0.5 mL·min^−1^/19 min	79.7–98.8%	19–70.1/27.6–94.8	<9.5%/<11%
[71]	LC-MS/MS/ESI+/SRM	Agella Durashell RPcolumn 100 mm × 2.1 mm, 5 μm + guard column with C18 (4.0 mm × 3.0 mm, 5 μm)/no information	(A)—0.1% FA in water (B)—0.1% FA in ACN	0.3 mL·min^−1^/12 min	27–74%	30/75	<16%/<11.1%
[54]	HPLC-MS/MS/ESI+/MRM	Kinetex biphenyl 5 µm, 2.1 mm × 50 mm + guard column (biphenyl, 2.0 mm × 4.0 mm)/30 °C	(A)—0.1% FA in water (B)—0.1% FA in ACN	0.4 mL·min^−1^/24 min	73.58–115.21%	6.5–34.1/4.1–16.4	<14%/<24%
[70]	LC-MS/MS/ESI+/MRM	Kinetex Biphenyl column (50 mm × 2.1 mm i.d., 2.6 μm)/no information	(A)—0.1% FA in ACN (B)—0.1% FA in water	0.2 mL·min^−1^/18 min	63.1–107.5%	5–20/15–50	<11.9%/<10.2%
[63]	HPLC-Q/Orbitrap MS/ESI+/(full MS ddMS^2^)	Obelisc R column (2.1 mm × 100 mm, 5 μm)/no information	(A)—0.1% FA in water with 2 mmol/L of ammonium acetate (B)—0.1% FA in ACN with 2 mmol/L of ammonium acetate	0.3 mL·min^−1^/26 min	74.9–94.3%	25/50	<15%
[77]	UHPLC-MS/MS/ESI+/MRM	Kinetex Biphenyl column, 100 mm × 2.1 mm, 2.6 μm/28 °C	(A)—0.1% FA in water (B)—0.1% FA in MeOH	0.35 mL·min^−1^/17 min	65.3–105.3%	No information/126–427,000	<11.2%/<32.6%
[69]	HPLC-MS/MS/ESI− and ESI+/sMRM	Gemini HPLC C18-column (5 μm 150 × 4.6 mm) + C18 security guard cartridge (4 mm × 3 mm i.d.)/25 °C	(A)—5 mM ammonium acetate buffer in MeOH/water/acetic acid (10/89/1, *v*/*v*/*v*) (B)—(97/2/1, *v*/*v*/*v*)	1 mL·min^−1^/21.5 min	60.5–79.35%	0.5–17.2/1.1–57.3	<8.2%/≤20% for the majority
[62]	UHPLC-MS/MS/ESI− and ESI+/MRM	Kinetex biphenyl column (100 mm × 2.1 mm, 2.6 µm) + guard column X-Bridge C18 (150 mm × 2.1 mm, 5 µm)/30 °C	(A)—0.2% FA in water (B)—0.2% FA in ACN	0.35 mL·min^−1^/8.5 min	88.6–107.8%	No information	<7.182%/<23.30%
[75]	HPLC-MS/MS/ESI− and ESI+/MRM	Imtakt Unison UK-C18 UP column (150 mm ×2.0 mm, 3.0 μm)/40 °C	(A)—Water with 5 mM ammonium formate and 0.1% FA (B)—MeOH with 5 mM ammonium formate and 0.1% FA	0.3 mL·min^−1^/30 min	70–118.66%	No information/20–100	<21.49%
[68]	HPLC-MS/MS/ESI− and ESI+/MRM	Imtakt Unison UK-C18 column (150 mm 3.0 mm, 3.0 μm, 120 Å)/40 °C	(A)—Water with 5 mM ammonium formate and 0.1% FA (B)—ACN with 5 mM ammonium formate and 0.1% FA	0.3 mL·min^−1^/30 min	70.04–119.94%	4–80/10–200	<17.30%/<27.19%
[80]	HPLC-MS/MS/ESI− and ESI+/sMRM	Gemini C18-column, 150 mm × 4.6 mm i.d., 5 μm + C18 security guard cartridge, 4 mm × 3 mm i.d./no information	(A)—5 mM ammonium acetate in MeOH/water/acetic acid (10/89/1, *v*/*v*/*v*) (B)—(97/2/1, *v*/*v*/*v*)	1 mL·min^−1^/20.5 min	No information	No information/10–50 for 80% of analytes	≤20% for the majority
[66]	LC-MS/MS/ESI+/MRM	Waters Acquity BEH (100 mm × 2.1 mm, 1.7 with the respective μm particle size) + pre-column (Acquity BEH VanGuard, 5 mm × 2.1 mm, 1.7 µm)/30 °C	(A)—water with 0.065% HFBA (*v*/*v*) (B)—ACN	No information/21 min	63–103%	0.5–4.6/2.6–86	<7.2%/<14%
[74]	UHPLC-LTQ/Orbitrap HRMS/ESI− and ESI+/data-dependent acquisition)	Reversed phase Hypersil GOLD analytical column (50 mm × 2.1 mm, 1.9 μm)/no information	(A)—water with 0.1% FA (B)—MeOH with 0.1% FA	0.25 mL·min^−1^/10 min	70.5–120.2%	0.5–15/2–50	<10.7/<13.1
[67]	UPLC-MS/MS/ESI+/MRM	Waters Peptide BEH C18 column (100 mm × 2.1 mm, 1.7 μm)/35 °C	(A)—0.1% FA in water (B)—ACN	0.3 mL·min^−1^/10 min	80.7–108.4%	2.2–6.0/7.2–20	<12.7/<15.7
[65]	UHPLC-MS/MS/ESI+/MRM	Thermo BDS HYPERSIL C18 column (2.1 mm ×100 mm, 2.4 μm)/35 °C	(A)—0.1% FA in water (B)—ACN	0.3 mL·min^−1^/8 min	85.2–100.3%	10/20	<14.2/<10.6

### 3.3. Detection Conditions

Almost all studies used the LC-MS/MS technique, except for the study [64], which used the LC-MS technique, whose mode of operation was selected ion monitoring (SIM), in which they selected the precursor ion for each tetracycline. The study by [71] opted for selected reaction monitoring (SRM) mode, in which they identified the most abundant fragments. Most used multiple reaction monitoring (MRM) mode, in which the most intense product ions are normally used for quantification and the second for confirmation. However, other studies, such as the study by [63], which used the HPLC-Q/Orbitrap MS technique, used the full MS-data dependent MS/MS (full MS ddMS2) mode, based on the most abundant ions. In the study by [74], the operating mode is data-dependent acquisition, in which selection is performed based on a list of precursor masses. The studies by [69,80] used scheduled multiple reaction monitoring (sMRM), in which each transition is monitored at an optimised time close to the retention time.

Most studies of multi-detection of various classes of veterinary drugs used positive and negative electrospray ionisation (ESI). In the study [73], it was found that the addition of 0.05% FA to the reconstitution solution increased the peak area of many analytes in ESI+, but the use of FA in ESI- was found to be unfavourable.

Regarding chromatographic columns, almost all are reverse-phase columns, except for the study [63] as they found that aminoglycosides, being polar, were not retained in the reverse-phase column. The study [66] also highlighted this difference and found good results when using a reverse-phase column with ionic pair and heptafluorobutyric acid (HFBA) in mobile phase A.

Most studies used a low percentage of FA, such as 0.04% or 0.1% in water, as the mobile phase A, as this improves sensitivity and peak resolution. However, some studies add ammonium acetate to control the pH to maintain acidity. In the study by [79] for ESI mode, they used water with 5 mM ammonium acetate as mobile phase A. Similarly, in the study by [63], they used 0.1% FA in water with 2 mmol/L of ammonium acetate as mobile phase A in ESI+ mode. In the study by [69,80], they used a solution of 5 mM ammonium acetate buffer in MeOH/water/acetic acid (10/89/1, *v*/*v*/*v*) for mobile phase A.

Similarly, the study [75] describes that to avoid suppression or intensification of the signal from antibiotics such as cephalosporins, a low pH is required for the mobile phase, for which they also used a buffer, 5 mM ammonium formate, and 0.1% FA in water. Similarly, in the study [68], which validated a multi-detection method, including cephalosporin antibiotics, they also used the same mobile phase A.

For mobile phase B, the studies maintain the chemical composition of mobile phase A, but change the most abundant solvent, usually water, to MeOH or ACN.

In the validated method [65] for the detection of macrolides, they found that for mobile phase B, MeOH produced irregular peaks, whereas ACN produced sharp peaks. Also, in the study [62], they obtained better chromatograms, i.e., better signal-to-noise ratio with ACN. In the study by [54], they confirm that 0.1% FA with ACN obtained better results when compared to MeOH, 0.1% FA with MeOH and ACN.

Only the study by [72] used isocratic elution, while the other authors used gradient elution.

### 3.4. Validation Process

The studies [54,62,64,70,72,73,78,79] validated the method in accordance with European Commission Decision 2002/657/EC [81]. Studies such as [68,75] have been validated in accordance with the Ministry of Food and Drug Safety’s Guidelines for standard procedures for preparing test methods for food. The most recent study [65] validated according to Regulation (EU) 2021/808 [82], which repeals and amends Decision 2002/657/EC, but is specific to the performance of analytical methods for residues of pharmacologically active substances used in food of animal origin. In contrast, studies such as [67,69,74,80] were validated in accordance with the SANTE Directive, the most recent being 11312/2021 [83], which contains procedures for validating methods for analysing pesticide residues in feed. They used this directive due to the absence of regulations with procedures for validation in matrices as complex as animal feed. The remaining studies were validated internally by evaluating parameters such as linearity, limit of detection (LOD), limit of quantification (LOQ), precision (intra- and inter-day), accuracy, matrix effects, and specificity.

According to Directive SANTE 11312/2021, the range 60–140% for recoveries, as well as repeatability relative standard deviation (RSDr) and reproducibility relative standard deviation (RSDR) ≤ 20%, are suitable for routine analyses. Of the validated methods for multi-detection of various classes of veterinary drugs, including antibiotics, those with the best recoveries between 60–140% were the studies by [68,69,74,75,77]. However, the study by [77] obtained a very high limit of quantification (LOQ) compared to Commission Delegated Regulation (EU) 2024/1229 [84], which establishes maximum LOQs for the identification of each antibiotic in non-target animal feed. In the study by [69], there is a low percentage of veterinary medicines that exceed the relative standard deviation (RDS) value of >20%. Similarly, the study of [68] presented an RSDR > 20%, but of the analytes it validated, LOQs were obtained in accordance with Commission Delegated Regulation (EU) 2024/1229.

Furthermore, the study by [75] shows RDS > 20% and LOQs higher than those defined in Commission Delegated Regulation (EU) 2024/1229 for the sulphonamide and pleuromutilin classes. Finally, the study by [74] showed not only good recoveries and low LOQs (2–50 µg/kg) in accordance with Commission Delegated Regulation (EU) 2024/1229, but also RSDR and RSDr values lower than 20%.

## 4. Conclusions

The inappropriate use of antibiotics as growth promoters in intensive animal production poses a risk to food safety and contributes to antimicrobial resistance, a public health concern. It is therefore essential to guarantee the control of animal feed in order to ensure compliance with European legislation.

The main challenge for detecting veterinary drugs in animal feed is the wide variety of raw materials, which causes the components to compete with the analytes, potentially leading to signal suppression or intensification. Also, the composition of feed varies considerably depending on the type of animal, which requires adaptation of extraction and purification procedures. For this reason, calibration is performed with the fortified matrix to minimise the effects of the matrix. However, some studies have used internal standards to correct deviations during extraction and purification, which makes the multiple detection method costly, as it requires several internal standards.

Although the study [74] shows good results for recoveries, LOD, LOQ, RSD_R_ and RSDr, most of the validated compounds are not listed in the table of Commission Delegated Regulation (EU) 2024/1229. For this reason, it is not possible to determine what the LOQ should be for the remaining analytes, as they are prohibited substances and no concentration is permitted. Therefore, as a future prospect, further regulation will be necessary to establish new maximum levels of cross-contamination of other veterinary medicinal products in non-target animal feed.

In short, it is essential to validate a method for the multi-detection of veterinary medicinal products, in particular antibiotics, which can achieve LOQs lower than or equal to the LOQs in the table in Commission Delegated Regulation (EU) 2024/1229. In this way, these procedures described serve as a basis for the development of a future method and are a starting point for a new multiple detection approach using UHPLC-MS/MS and UHPLC-ToF-MS.

## Data Availability

No new data were created or analyzed in this study.
